# Supplementary material for: An economic evaluation of Wolbachia deployments for dengue control in Vietnam
Source: PLoS Negl Trop Dis. 2023 May 30;17(5):e0011356. doi: 10.1371/journal.pntd.0011356 (PMC10256143; doi:10.1371/journal.pntd.0011356)
Supplement: S1 Checklist — (DOCX) [file pntd.0011356.s002.docx]

| **The CHEERS 2022 checklist** | | | |
| --- | --- | --- | --- |
| Section/topic | Item No | Guidance for reporting | Reported in section |
| **Title** | | |  |
| Title | 1 | Identify the study as an economic evaluation and specify the interventions being compared. | Title |
| **Abstract** | | |  |
| Abstract | 2 | Provide a structured summary that highlights context, key methods, results, and alternative analyses. | Abstract |
| **Introduction** | | |  |
| Background and objectives | 3 | Give the context for the study, the study question, and its practical relevance for decision making in policy or practice. | Introduction |
| **Methods** | | |  |
| Health economic analysis plan | 4 | Indicate whether a health economic analysis plan was developed and where available. | Supporting information: Further methodological details |
| Study population | 5 | Describe characteristics of the study population (such as age range, demographics, socioeconomic, or clinical characteristics). | Methods: The selected settings and dengue incidence and the Supporting information: Project sites and stratification of dengue cases |
| Setting and location | 6 | Provide relevant contextual information that may influence findings. | Methods: The selected settings and dengue incidence and Table 1 |
| Comparators | 7 | Describe the interventions or strategies being compared and why chosen. | Methods: The economic evaluation |
| Perspective | 8 | State the perspective(s) adopted by the study and why chosen. | Methods: The economic evaluation and Box 1 |
| Time horizon | 9 | State the time horizon for the study and why appropriate. | Methods: The economic evaluation |
| Discount rate | 10 | Report the discount rate(s) and reason chosen. | Methods: The economic evaluation |
| Selection of outcomes | 11 | Describe what outcomes were used as the measure(s) of benefit(s) and harm(s). | Methods: Effectiveness of the Wolbachia deployments, Box 1 and Supporting information |
| Measurement of outcomes | 12 | Describe how outcomes used to capture benefit(s) and harm(s) were measured. | Methods: Effectiveness of the Wolbachia deployments, Box 1 and Supporting information |
| Valuation of outcomes | 13 | Describe the population and methods used to measure and value outcomes. | Methods: Effectiveness of the Wolbachia deployments, Methods: Health burden and economic burden of dengue and Supporting information |
| Measurement and valuation of resources and costs | 14 | Describe how costs were valued. | Methods: Costs of the Wolbachia deployments and Supporting information |
| Currency, price date, and conversion | 15 | Report the dates of the estimated resource quantities and unit costs, plus the currency and year of conversion. | Methods: The economic evaluation |
| Rationale and description of model | 16 | If modelling is used, describe in detail and why used. Report if the model is publicly available and where it can be accessed. | Methods: The economic evaluation |
| Analytics and assumptions | 17 | Describe any methods for analysing or statistically transforming data, any extrapolation methods, and approaches for validating any model used. | NA |
| Characterising heterogeneity | 18 | Describe any methods used for estimating how the results of the study vary for subgroups. | Supporting information: Further methodological details |
| Characterising distributional effects | 19 | Describe how impacts are distributed across different individuals or adjustments made to reflect priority populations. | Supporting information: Further methodological details |
| Characterising uncertainty | 20 | Describe methods to characterise any sources of uncertainty in the analysis. | Methods: The economic evaluation and Table 3 |
| Approach to engagement with patients and others affected by the study | 21 | Describe any approaches to engage patients or service recipients, the general public, communities, or stakeholders (such as clinicians or payers) in the design of the study. | Supporting information |
| **Results** | | |  |
| Study parameters | 22 | Report all analytic inputs (such as values, ranges, references) including uncertainty or distributional assumptions. | Table 2 and Supporting information |
| Summary of main results | 23 | Report the mean values for the main categories of costs and outcomes of interest and summarise them in the most appropriate overall measure. | Table 3 and results |
| Effect of uncertainty | 24 | Describe how uncertainty about analytic judgments, inputs, or projections affect findings. Report the effect of choice of discount rate and time horizon, if applicable. | Results: Sensitivity analysis |
| Effect of engagement with patients and others affected by the study | 25 | Report on any difference patient/service recipient, general public, community, or stakeholder involvement made to the approach or findings of the study | NA |
| **Discussion** | | |  |
| Study findings, limitations, generalisability, and current knowledge | 26 | Report key findings, limitations, ethical or equity considerations not captured, and how these could affect patients, policy, or practice. | Discussion and Supporting information |
| **Other relevant information** | | | |
| Source of funding | 27 | Describe how the study was funded and any role of the funder in the identification, design, conduct, and reporting of the analysis | Funding statement |
| Conflicts of interest | 28 | Report authors conflicts of interest according to journal or International Committee of Medical Journal Editors requirements. | Conflicts of interest statement |
