# Supplementary material for: An economic evaluation of Wolbachia deployments for dengue control in Vietnam
Source: PLoS Negl Trop Dis. 2023 May 30;17(5):e0011356. doi: 10.1371/journal.pntd.0011356 (PMC10256143; doi:10.1371/journal.pntd.0011356)
Supplement: S1 Table — (DOCX) [file pntd.0011356.s003.docx]

| **S1 Table: Baseline burden of dengue across the study settings** | | | | | | | | | | | | |
| --- | --- | --- | --- | --- | --- | --- | --- | --- | --- | --- | --- | --- |
| **Setting** | **Baseline annual epidemiology burden** | | | | | | **Baseline annual economic burden (2020 US$ prices)** | | | | | |
|  | **Sought no formal treatment** | **Sought formal treatment- outpatient** | **Sought formal treatment - hospitalized** | **Total number of cases** | **Fatal cases** | **Baseline DALYs burden** | **Cases that sought no formal treatment** | **Outpatient cases** | **Hospitalized cases** | **Fatal cases** | **Government’s current dengue prevention and control activities** | **Total economic burden** |
| Hồ Chí Minh | 103,574 | 37,649 | 20,359 | 161,582 | 18.8 | 6,085 | 1,793,724 | 2,598,869 | 4,556,207 | 1,598,047 | 315,602 | 10,818,388 |
| Hà Nội | 74,228 | 26,981 | 14,591 | 115,800 | 13.5 | 4,361 | 1,285,500 | 1,862,520 | 3,265,276 | 1,145,265 | 220,268 | 7,879,041 |
| Đà Nẵng | 7,903 | 2,873 | 1,554 | 12,329 | 1.4 | 464 | 136,870 | 198,306 | 347,661 | 121,939 | 37,387 | 852,752 |
| Cần Thơ | 12,413 | 4,512 | 2,440 | 19,366 | 2.3 | 729 | 214,980 | 311,478 | 546,067 | 191,528 | 34,964 | 1,304,675 |
| Thuận An | 8,401 | 3,054 | 1,651 | 13,106 | 1.5 | 494 | 145,485 | 210,789 | 369,545 | 129,614 | 18,280 | 873,741 |
| Dĩ An | 6,691 | 2,432 | 1,315 | 10,439 | 1.2 | 393 | 115,883 | 167,900 | 294,353 | 103,242 | 15,800 | 695,953 |
| Thủ Dầu Một | 4,994 | 1,815 | 982 | 7,791 | 0.9 | 293 | 86,489 | 125,310 | 219,688 | 77,054 | 9,710 | 518,416 |
| Biên Hòa | 9,028 | 3,282 | 1,775 | 14,084 | 1.6 | 530 | 156,346 | 226,524 | 397,131 | 139,290 | 35,399 | 959,264 |
| Nha Trang | 2,573 | 935 | 506 | 4,014 | 0.5 | 151 | 44,558 | 64,558 | 113,180 | 39,697 | 14,691 | 289,268 |
| Vũng Tàu | 2,933 | 1,066 | 577 | 4,576 | 0.5 | 172 | 50,794 | 73,594 | 129,021 | 45,253 | 9,283 | 317,141 |
| **Total** | **232,738** | **84,599** | **45,749** | **363,086** | **42** | **13,674** | **4,030,630** | **5,839,849** | **10,238,129** | **3,590,929** | **809,105** | **24,508,641** |
