# Supplementary material for: An economic evaluation of Wolbachia deployments for dengue control in Vietnam
Source: PLoS Negl Trop Dis. 2023 May 30;17(5):e0011356. doi: 10.1371/journal.pntd.0011356 (PMC10256143; doi:10.1371/journal.pntd.0011356)
Supplement: S2 Table — (DOCX) [file pntd.0011356.s004.docx]

| **S2 Table: Comparison of the projected number of hospitalized cases to Ministry of Health data** | | | | |
| --- | --- | --- | --- | --- |
|  | | **Projected number of hospitalized cases** | **The average number of reported hospitalized cases – Ministry of Health data (2016-2019)** | **Ratio of the projected to reported hospitalized cases** |
| Hồ Chí Minh | | 20,359 | 11,612 | 1.75 |
| Hà Nội | | 14,591 | 10,619 | 1.37 |
| Đà Nẵng | | 1,554 | 4,231 | 0.37 |
| Cần Thơ | | 2,440 | 954 | 2.56 |
| Thuận An | | 1,651 | 2,110 | 0.78 |
| Dĩ An | | 1,315 | 1,516 | 0.87 |
| Thủ Dầu Một | | 982 | 1,504 | 0.65 |
| Biên Hòa | | 1,775 | 1,757 | 1.01 |
| Nha Trang | | 506 | 1,568 | 0.32 |
| Vũng Tàu | | 577 | 884 | 0.65 |
| **Total** |  | 45,749 | 36,754 | 1.24 |
