# Supplementary material for: An economic evaluation of Wolbachia deployments for dengue control in Vietnam
Source: PLoS Negl Trop Dis. 2023 May 30;17(5):e0011356. doi: 10.1371/journal.pntd.0011356 (PMC10256143; doi:10.1371/journal.pntd.0011356)
Supplement: S3 Table — (DOCX) [file pntd.0011356.s005.docx]

| **S3 Table: Base case projected total cost and impact of the** ***Wolbachia* deployments (2020 US$ prices)** | | | | | | | | | | |
| --- | --- | --- | --- | --- | --- | --- | --- | --- | --- | --- |
| **Setting** | **Total cost of the intervention (US$)** | **DALYs averted** | **Averted cases that sought no formal treatment** | **Averted outpatient cases** | **Averted hospitalized cases averted** | **Total number of cases averted** | **Total cost of illness averted (US$)^1^** | **Total economic burden averted (US$)^2^** | **Breakeven year (from the start of the intervention)** | **Societal benefit-cost ratio** |
| Hồ Chí Minh | 76,002,063 | 68,215 | 1,776,452 | 645,730 | 349,193 | 2,771,698 | 129,184,363 | 132,125,986 | 13 | 1.74 |
| Hà Nội | 53,044,085 | 48,888 | 1,273,122 | 462,773 | 250,255 | 1,986,380 | 92,581,972 | 96,053,767 | 13 | 1.81 |
| Đà Nẵng | 9,003,297 | 5,205 | 135,552 | 49,272 | 26,645 | 211,494 | 9,857,396 | 10,377,131 | 19 | 1.15 |
| Cần Thơ | 8,420,004 | 8,176 | 212,910 | 77,392 | 41,851 | 332,191 | 15,482,906 | 15,922,973 | 12 | 1.89 |
| Thuận An | 4,402,016 | 5,533 | 144,084 | 52,374 | 28,322 | 224,807 | 10,477,887 | 10,676,213 | 10 | 2.43 |
| Dĩ An | 3,804,998 | 4,407 | 114,767 | 41,717 | 22,560 | 179,065 | 8,345,934 | 8,503,830 | 11 | 2.23 |
| Thủ Dầu Một | 2,338,347 | 3,289 | 85,656 | 31,135 | 16,837 | 133,644 | 6,228,921 | 6,335,899 | 9 | 2.71 |
| Biên Hòa | 8,524,624 | 5,946 | 154,840 | 56,284 | 30,437 | 241,588 | 11,260,043 | 11,693,085 | 16 | 1.37 |
| Nha Trang | 3,537,894 | 1,695 | 44,129 | 16,041 | 8,674 | 68,851 | 3,209,056 | 3,504,532 | - | 0.99 |
| Vũng Tàu | 2,235,582 | 1,932 | 50,305 | 18,286 | 9,888 | 78,488 | 3,658,206 | 3,858,388 | 13 | 1.73 |
| **Total** | **171,312,910** | **153,285** | **3,991,817** | **1,451,004** | **784,663** | **6,228,208** | **290,286,683** | **299,051,805** | **-** | **1.75** |
| *^1^ The cost of illness is the cost a speciﬁc disease or condition imposes on society (i.e the direct costs and productivity costs associated with dengue cases).*  *^2^ The economic burden includes the cost of illness but also the costs associated with government’s current dengue prevention and control activities.* | | | | | | | | | | |
