# Supplementary material for: An economic evaluation of Wolbachia deployments for dengue control in Vietnam
Source: PLoS Negl Trop Dis. 2023 May 30;17(5):e0011356. doi: 10.1371/journal.pntd.0011356 (PMC10256143; doi:10.1371/journal.pntd.0011356)
Supplement: S4 Table — (DOCX) [file pntd.0011356.s006.docx]

| **S4 Table: Base case setting specific cost-effectiveness ratios (2020 US$ prices)** | | | | | | | |
| --- | --- | --- | --- | --- | --- | --- | --- |
| **Setting** | **Projected incidence per 100,000 population^1^** | **Gross cost-effectiveness ratio** | **Incremental cost-effectiveness ratio - health care provider perspective** | **Incremental cost-effectiveness ratio - health sector perspective** | **Incremental cost-effectiveness ratio - societal perspective** | **Incremental cost-effectiveness ratio - societal perspective (excluding the productivity gains related to prevented excess mortality)** |  |
| Hồ Chí Minh | 1,797 | 1,114 | 776 | 430 | -780 | -493 |  |
| Hà Nội | 1,438 | 1,085 | 719 | 373 | -809 | -522 |  |
| Đà Nẵng | 1,087 | 1,730 | 1,335 | 989 | -164 | 123 |  |
| Cần Thơ | 1,568 | 1,030 | 681 | 335 | -864 | -577 |  |
| Thuận An | 2,198 | 796 | 465 | 119 | -1,098 | -811 |  |
| Dĩ An | 2,199 | 863 | 533 | 187 | -1,030 | -743 |  |
| Thủ Dầu Một | 2,423 | 711 | 383 | 38 | -1,183 | -896 |  |
| Biên Hòa | 1,334 | 1,434 | 1,066 | 720 | -460 | -173 |  |
| Nha Trang | 950 | 2,088 | 1,618 | 1,273 | 194 | 481 |  |
| Vũng Tàu | 1,281 | 1,157 | 759 | 413 | -736 | -450 |  |
| **Overall** | **1,627** | **1,118** | **708** | **420** | **-776** | **-546** |  |
| *^1^ Based on the projected case numbers and the total population within the administrative district boundary (Table 1 of the main text).* | | | | | | | |
