# Supplementary material for: An economic evaluation of Wolbachia deployments for dengue control in Vietnam
Source: PLoS Negl Trop Dis. 2023 May 30;17(5):e0011356. doi: 10.1371/journal.pntd.0011356 (PMC10256143; doi:10.1371/journal.pntd.0011356)
Supplement: S5 Table — (DOCX) [file pntd.0011356.s007.docx]

| **S5 Table:** **Results of the sensitivity analysis (2020 US$ prices)** | | | | | | | | | | | | | | | | | |
| --- | --- | --- | --- | --- | --- | --- | --- | --- | --- | --- | --- | --- | --- | --- | --- | --- | --- |
|  | **Base case results** | **Using the confidence interval on the baseline incidence of dengue cases** | | **Assuming an alternative breakdown of the type of care dengue cases receive** | **DALY weights - accounting for only acute symptoms** | **Using the confidence intervals for the cost of illness of non-fatal cases** | | **Varying the assumed annual growth in case numbers (0-5%)** | | **Varying the assumed effectiveness of the Wolbachia deployments (65%-85%)** | | **Varying the assumed duration of the benefits (10-25 years)** | | **Assuming the long-term monitoring phase lasts 20 years** | **Using a 0% discount rate for the health effects** | **Using a 6% discount rate for the costs** |  |
|  |  | **Lower** | **Higher** |  |  | **Lower** | **Higher** | **Lower** | **Higher** | **Lower** | **Higher** | **Lower** | **Higher** |  |  |  |  |
| Baseline DALY burden | 13,674 | 11,454 | 16,627 | 13,745 | 6,417 | 13,674 | 13,674 | 13,674 | 13,674 | 13,674 | 13,674 | 13,674 | 13,674 | 13,674 | 13,674 | 13,674 |  |
| Number of DALYs averted (thousands) | 153 | 128 | 186 | 154 | 64 | 153 | 153 | 136 | 243 | 133 | 174 | 84 | 184 | 153 | 153 | 153 |  |
| Total cost of the intervention (million) | 171 | 171 | 171 | 171 | 171.31 | 171 | 171 | 171 | 171 | 171 | 171 | 171 | 171 | 120 | 125 | 174 |  |
| Cost per person reached | 8.56 | 8.56 | 8.56 | 8.56 | 8.56 | 8.56 | 8.56 | 8.56 | 8.56 | 8.56 | 8.56 | 8.56 | 8.56 | 5.99 | 6.25 | 8.68 |  |
| Societal benefit-cost ratio | 1.75 | 1.47 | 2.11 | 2.69 | 1.75 | 1.42 | 2.16 | 1.55 | 2.74 | 1.52 | 1.97 | 0.95 | 2.09 | 2.49 | 2.39 | 1.72 |  |
| Gross cost-effectiveness ratio | 1,118 | 1,334 | 919 | 1,111 | 2,660 | 1,118 | 1,118 | 1,264 | 705 | 1,290 | 986 | 2,050 | 933 | 783 | 816 | 1,133 |  |
| ICER - health sector perspective | 420 | 625 | 231 | -45 | 999 | 557 | 263 | 558 | 28 | 583 | 295 | 1349 | 239 | 85 | 118 | 435 |  |
| ICER - societal perspective (excluding the productivity gains related to prevented excess mortality) | -546 | -341 | -735 | -1,592 | -1,301 | -177 | -1,013 | -408 | -938 | -383 | -671 | 383 | -727 | -881 | -848 | -531 |  |
| *See Table 2 of the main text for parameter ranges.*  *WMP; World Mosquito Program, ICER; Incremental cost-effectiveness ratio.*  *Negative ratios (“Cost savings”) in the case indicate that the economic benefits of the health intervention relative to the comparator outweighed the cost of the intervention. Note that these “Cost savings” include non-fiscal costs* | | | | | | | | | | | | | | | | | |
