# Supplementary material for: An economic evaluation of Wolbachia deployments for dengue control in Vietnam
Source: PLoS Negl Trop Dis. 2023 May 30;17(5):e0011356. doi: 10.1371/journal.pntd.0011356 (PMC10256143; doi:10.1371/journal.pntd.0011356)
Supplement: S6 Table — (DOCX) [file pntd.0011356.s008.docx]

| **S6 Table:** **Setting specific cost-effectiveness ratios – when assuming only 10 years of benefits (2020 US$ prices)** | | | | | | | |
| --- | --- | --- | --- | --- | --- | --- | --- |
| **Setting** | **Gross cost-effectiveness ratio** | | **Incremental cost-effectiveness ratio - health care provider perspective** | **Incremental cost-effectiveness ratio - health sector perspective** | | **Incremental cost-effectiveness ratio - societal perspective** | **Incremental cost-effectiveness ratio - societal perspective (excluding the productivity gains related to prevented excess mortality)** |
| Hồ Chí Minh | | 2,044 | 1,704 | | 1,358 | 150 | 437 |
| Hà Nội | | 1,990 | 1,621 | | 1,275 | 97 | 384 |
| Đà Nẵng | | 3,173 | 2,773 | | 2,427 | 1,279 | 1,566 |
| Cần Thơ | | 1,889 | 1,538 | | 1,192 | -4 | 282 |
| Thuận An | | 1,460 | 1,127 | | 781 | -434 | -147 |
| Dĩ An | | 1,584 | 1,251 | | 905 | -310 | -23 |
| Thủ Dầu Một | | 1,304 | 975 | | 629 | -590 | -303 |
| Biên Hòa | | 2,630 | 2,259 | | 1,913 | 736 | 1,023 |
| Nha Trang | | 3,830 | 3,352 | | 3,006 | 1,936 | 2,223 |
| Vũng Tàu | | 2,123 | 1,719 | | 1,373 | 229 | 516 |
| **Overall** | | **2,050** | **1,635** | | **1,349** | **156** | **383** |
|  | | | | | | | |
