# Supplementary material for: An economic evaluation of Wolbachia deployments for dengue control in Vietnam
Source: PLoS Negl Trop Dis. 2023 May 30;17(5):e0011356. doi: 10.1371/journal.pntd.0011356 (PMC10256143; doi:10.1371/journal.pntd.0011356)
Supplement: S7 Table — (DOCX) [file pntd.0011356.s009.docx]

| **S7 Table: The cost-effectiveness and cost benefit of the Wolbachia deployments for different intervention costs** | | | | | | | | |
| --- | --- | --- | --- | --- | --- | --- | --- | --- |
|  | **Average discounted cost per person covered (US$)** | | | | | | | |
|  | **US$3** | **US$5** | **US$8.56**  **(Base case)** | **US$12.00** | **US$3** | **US$5** | **US$8.56**  **(Base case)** | **US$12.00** |
|  | **10 years of benefits** | | | | **20 years of benefits** | | | |
| Total cost | 60,059,504 | 100,099,173 | 171,369,785 | 240,238,016 | 60,059,504 | 100,099,173 | 171,369,785 | 240,238,016 |
| Gross cost-effectiveness ratio | 719 | 1,198 | 2,051 | 2,875 | 392 | 653 | 1,118 | 1,567 |
| Incremental cost-effectiveness ratio - health care provider perspective | 303 | 783 | 1,636 | 2,460 | -18 | 244 | 709 | 1,158 |
| Incremental cost-effectiveness ratio - health sector perspective | 18 | 497 | 1,350 | 2,174 | -306 | -45 | 420 | 869 |
| Incremental cost-effectiveness ratio - societal perspective | “Cost saving” (-1,175) | “Cost saving” (-696) | 157 | 981 | “Cost saving” (-1,502) | “Cost saving” (-1,241) | “Cost saving” (-776) | “Cost saving” (-327) |
| Incremental cost-effectiveness ratio - societal perspective (excluding the productivity gains related to prevented excess mortality) | “Cost saving” (-948) | “Cost saving” (-469) | 384 | 1,208 | “Cost saving (-1,272) | “Cost saving” (-1,011) | “Cost saving” (-546) | “Cost saving” (-97) |
| Societal benefit-cost ratio | 2.72 | 1.63 | 0.95 | 0.68 | 4.98 | 2.99 | 1.75 | 1.24 |
| *Negative ratios (“Cost savings”) in the case indicate that the economic benefits of the health intervention relative to the comparator outweighed the cost of the intervention. Note that these “Cost savings” include non-fiscal costs.* | | | | | | | | |
